# Association of placental histopathological findings with COVID-19 and its predictive factors

**DOI:** 10.61622/rbgo/2024AO03

**Published:** 2024-03-15

**Authors:** Zeena Helmi, Hadeel Al-badri

**Affiliations:** 1 Mustansiriyah University College of Medicine Department of Gynaecology and Obstetrics Baghdad Iraq Department of Gynaecology and Obstetrics, College of Medicine, Mustansiriyah University, Baghdad, Iraq.

**Keywords:** COVID-19, Coronavirus infections, Histopathology, Placenta

## Abstract

**Objective:**

The aims of the study are to describe the association of coronavirus disease (COVID-19) with the abnormal histopathological findings in human placenta and to highlight the potential predictors of these histopathological findings.

**Methods:**

A retrospective cohort study, held in two obstetric units from January 2021- 2022, 34 patients who were confirmed cases of COVID- 19 were followed up till the time of delivery as their placenta were sent for histopathology. Patients diagnosed with other viral infections, chorioamnionitis, or were known case of as pre-term or term pre labour rupture of membrans (PROM) were excluded as well as pre exisiting diabetes mellitus or pre-eclampsia. Data analysis were performed using STATA software version 16.

**Result:**

Specific histopatological findings (fetal vascular malperfusion, maternal vascular malperfusion, inflammatory pathology and thrombotic finding) were significantly high among 13 (38.2%) of the study group who got infected earlier in pregnancy (P<0.001). The period between the diagnosis of COVID-19 and the delivery significantly increases the odds of the presence of pathological findings by 2.75 times for each week the patients getting infected earlier.

**Conclusion:**

Association of abnormal placental histopathological findings with COVID-19 infection in pregnancy and the potential predictor for the occurrence of placental findings is the longer duration between the diagnosis of the infection and the delivery.

## Introduction

Since march 2020, when coronavirus was announced as a pandemic, researchers have worked to explore the effect of this novel virus on pregnancy.^(1)^ Many adverse pregnancy outcomes have been associated with this infection as miscarriage,^(2)^ pre-eclampsia, pre-term labor,^(3)^ intra-uterine fetal demise, and fetal and maternal morbidity and mortality.^(4)^

Coronaviruses are single-stranded RNA viruses that's one of their main receptors is angiotensin-converting enzyme 2 (ACE2) which critically important in hemodynamic maternal adaptaion, ensuring healthy pregnancy. During pregnancy, a drecrease in ACE2 expression has been linked to intrauterine growth restriction (IUGR) and Pre-eclampsia, Therefore, COVID-19 infection may lead to a compromise in the vascularization of placental, elevation in blood pressure, placental dysfunction that results in other adverse outcomes. A decline in placental ACE2 expression normally occurs during pregnancy. Important reductions in expression levels were found from the first to the second trimester until it became almost undetectable in the third trimester.^(1)^

The human placenta is the only physical connection between mother and fetus; it provides the surface area for maternal-fetal exchange to create an ideal environment for fetal growth and optimal development.^(5)^Detailed evaluation of the placenta after delivery aid in evaluating the disease process and identifying the solution for prevention and treatment.^(6,7)^ Many known viruses can cross the placenta, like the zika virus, cytomegaly virus, and rubella virus; however, till now, there has been no proven vertical transmission of the coronavirus.^(8)^

Histopathological assessment of the human placenta from mothers with coronavirus infection has been reported.^(9)^ However reports about these findings in different trimesters in pregnancy are limited and not well-known.^(10)^

A growing body of evidence confirmed a higher rate of placental arteriopathy and malperfuson of the maternal and fetal vasculature on histopathological examination,^(11)^ but no studies have correlated the effect of duration on placental histopathological findings.

Hence, this study aims to evaluate the histopathological findings of placenta in patients who had coronavirus infection in late second and third trimester of pregnancy from the onset of infection till the time of delivery.

## Methods

This is a retrospective cohort study carried out in two obstetric units in Iraq Baghdad, Yarmouk Teaching Hospital in Baghdad and Shaheed Fairouz Hospital in Wasit Province, Iraq, from January 2021 to January 2022; the College of Medicine/ University of Mustansiriyah approved the study IRB (131) and all patient signed informed consent.

Inclusion criteria: patients who attended the obstetric unit with a singleton pregnancy who had a confirmed laboratory diagnosis of SARS CoV-19 infections during the second half of pregnancy were included in the study and were followed up till time of delivery. Diagnosis of SARS-COV- 19 was confirmed by taking a swab from the nasopharynx and confirmed by qualitative real-time polymerase chain reaction (PCR). All the patients had a PCR during admission for SARS-CoV infection.

Exclusion criteria: pregnant women with unknown PCR SAR-CoV infection, patients with known other viral infection as chicken pox, and patients with a known case of chorioamnionitis as pre-term or term pre labour rupture of membrans (PROM).

**Figure 1:**
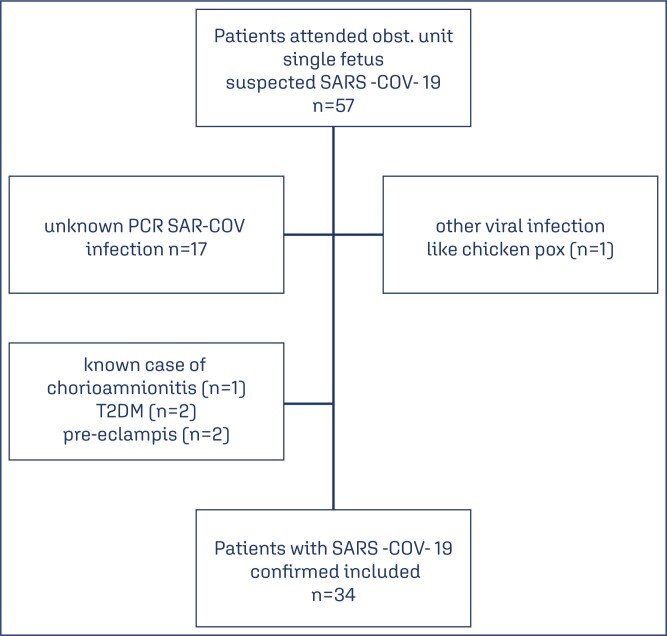
Study flowchart

Patients with history of pre exisiting diabetes mellitus and pre-eclampsia diagnosis were excluded from the study by clinical examination for hypertension in addition to protein urea and blood examination of thrombocytopenia and elevated liver enzyme (Figure 1).^(12)^

All patients were followed up till the time of delivery as their placentas were taken and sent for histopathological study in the Yarmouk teaching hospital within 24 hours; all placentas were examined under a standard protocol by two histopathologists who were double- blinded.

According to obstetric indications, all patients with COVID-19 PCR positive were included in the study after giving birth by vaginal or cesarean section. The placenta was fixated in 10% buffered formalin (should be added within 2 hours of collection) until sectioning was done and further processing.

Multiple sections were taken in 4 μm thickness from fetal and maternal sides in full placental thickness, two umbilical cords, one membrane, and two placental discs full thickness, including maternal and fetal surfaces. In addition to any grossly suspected lesion, representative specimens have collected a total of about 5 -8 sections and further stained with eosin-hematoxylin stain.

Microscopic evaluation was done for each placenta according to the Amsterdam placental group,^(13)^which was divided into four groups, including fetal vascular malperfusion (abnormal variable size fetal and chorionic villi), maternal vascular malperfusion (decidual arterio-pathy, Intervillous increase of fibrin and increased syncytial knots), third group inflammatory pathology (Lymphocytic villitis and intervillositis) and fourth group thrombotic findings (fibrinoid necrosis of maternal arteriole), as shown on figure.^(2–6)^

Maternal clinical and hematological findings were collected, including (age, BMI, gestational age at the time of infection, gestational age at the time of delivery, duration between infection and delivery, gravidity, parity, history of previous abortion, hemoglobin, platelets, white blood cells (WBC) level (at time of infection), lymphocytes, blood group and RH and admission to neonatal care unit and categorization according to the severity of covid infection according to National Institutes of Health guidelines for asymptomatic, mild, moderate, severe, or critical illness.^(14)^

Neonatal characteristics were reviewed, including birth weight, Apgar score at 1 and 5-minute, gestational age at time of delivery, whether term or pre-term, and admission to the neonatal care unit.

### The statistical analysis

Data entry, cleaning, and analysis were performed using STATA software version 16. The categorical data were presented with frequencies and percentages and the association with the main variable using Pearson's chi-square test.

The continuous data were tested for the normality of the distribution, and accordingly, some of the parameters (normally distributed) were presented by their average and standard deviation and compared between the two categories of the dependent variable (Presence or absence of placental histo-pathological findings) using Student's t-test. The significant parameters were measured for multicollinearity with a variable inflation factor of less than 10 as the acceptable level. Logistic regression analysis was used to refine the predictive parameters of the presence of placental histopathological findings. The confidence level used in these analyses was 95%. A P-value less than 0.05 was significant.

## Results

**Figure 2:**
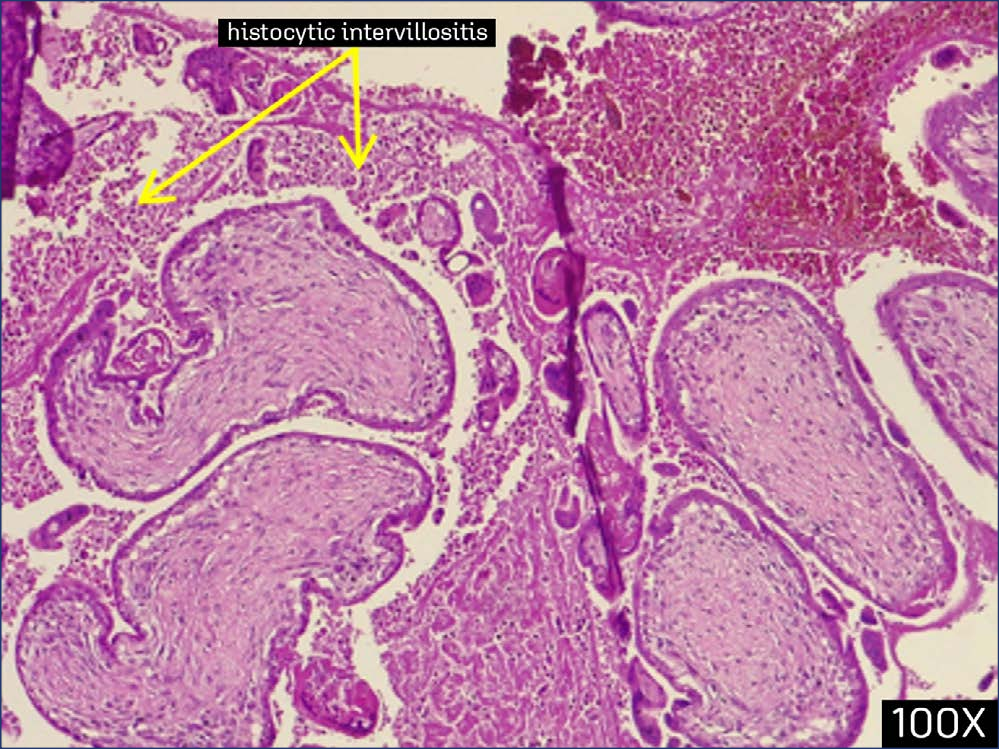
Chronic villitis and histocytic intervillositis (arrow) in the placenta (confirmed covid infection at 28 weeks, specimen collected after delievery at 37 weeks) (100x)

**Figure 3:**
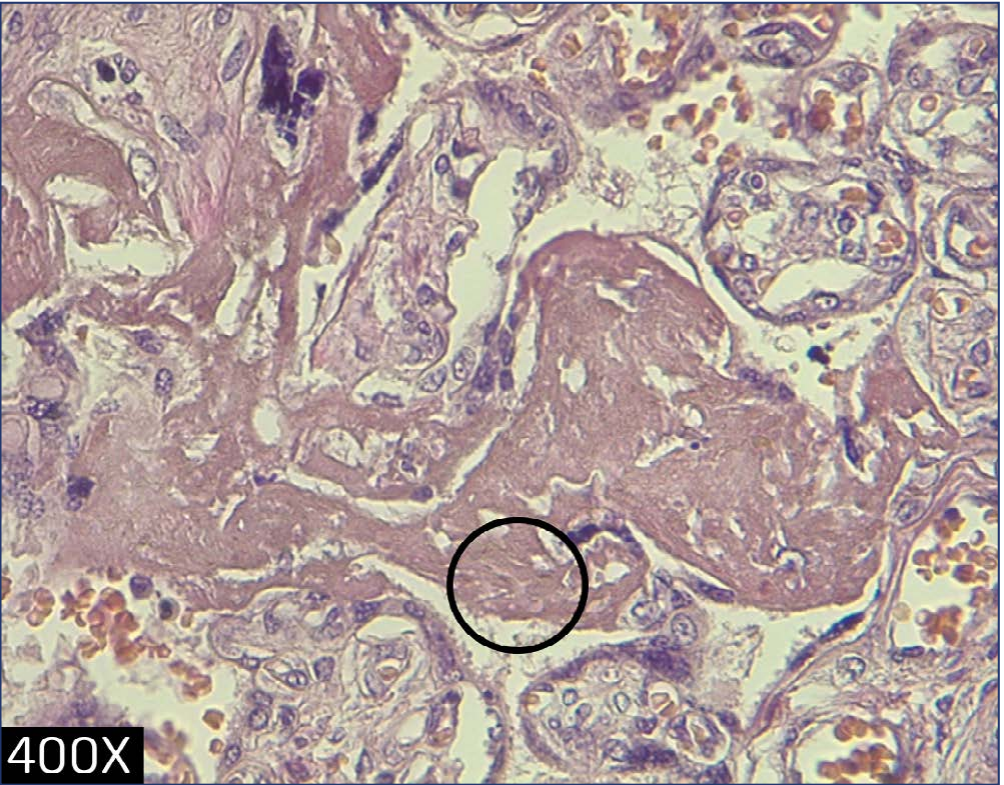
Perivillous fibrin deposition (circle) as result of maternal malperfusion, patient infected at 26 weeks (400x)

**Figure 4:**
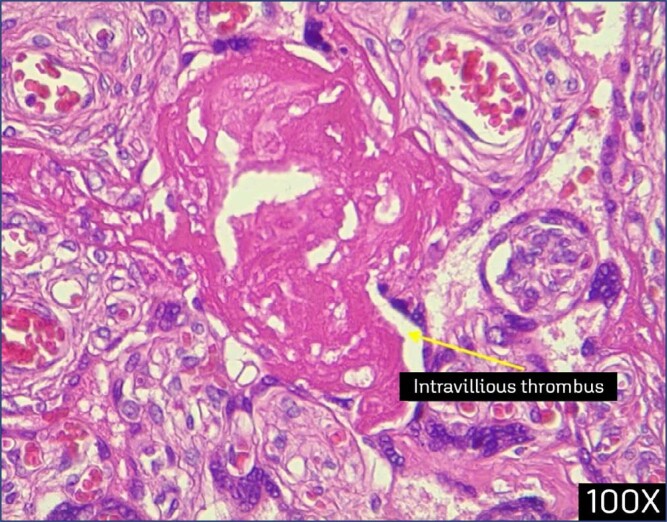
Cross section of placenta in a patient infected at 33 weeks of gestation, showing intervillous thrombosis (arrows) (x100)

**Figure 5:**
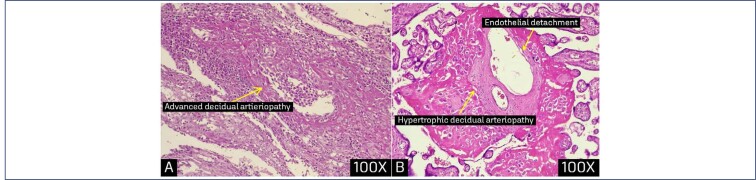
Cross section of a placenta in a COVID confimed case diagnosed at 31 weeks of gestation A: showing advanced decidual artieriopathy (x100), B: magnified picture of hypertrophic decidual arteriopathy with endothelial detachment (x100)

**Figure 6:**
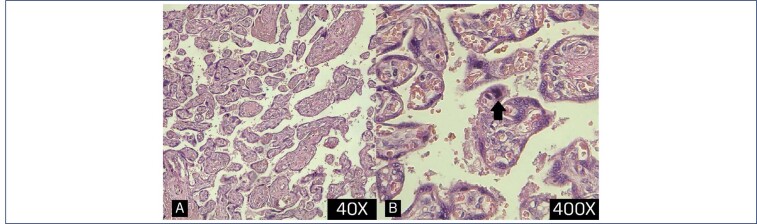
Cross section of a placenta in a COVID confimed case diagnosed at 31 weeks of gestation showing A: increase synctial knotting (x40), B: higher magnification (x400) of the same slide (arrow) syncytial knot

**Figure 7:**
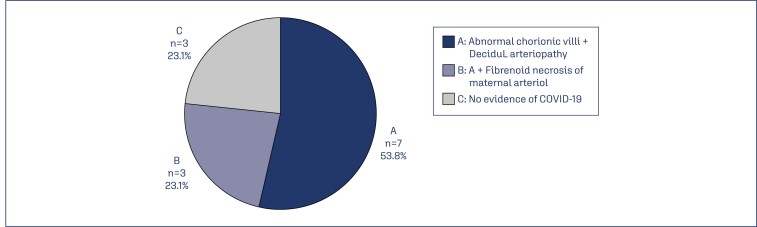
The main placentae histo-pathological findings among the studied patients (n=13)

The current study investigates the possible determinants of histopathological findings in the placenta among 34 patients who have been diagnosed with COVID-19 cases during their pregnancy. Findings appeared among 13 (38.2%) of the study group, with the details shown in figures 2–7.

The main maternal parameters were compared according to the presence of the placental pathological findings. There was no significant difference between the two groups regarding their age, body mass indices, and gestational age at delivery. On the other hand, those with significant placental findings were infected by COVID-19 late second (only 4 cases) and early third trimester (P<0.001), meaning a significantly longer period from infection to delivery. The white blood cells were significantly lower among the group with histopathological findings (P=0.006), while the lymphocytes and random blood glucose were significantly higher (P=0.013 and P=0.005), respectively. Maternal hemoglobin and platelets level did not show such significant differences. Other maternal factors did not show any association with histopathological findings. As shown in chart 1.

**Chart 1:** Comparison of maternal parameters according to the presence of placental pathological findings

Parameters	Placental pathological findings				p-value*
	Yes (n=13)		No (n=21)		
	Mean	SD	Mean	SD	
Maternal age (years)	28.74	5.46	28.03	6.54	0.735
Body mass index (Kilograms/meter^2^)	27.69	4.43	27.92	5.03	0.886
Timing of infection during pregnancy (Weeks)	28.15	3.76	35.95	1.40	<0.001*
Gestational age at delivery (mean in Weeks)	37.26	1.62	37.01	1.91	0.686
COVID-19 diagnosis to delivery (Weeks)	9.2	4.4	1.1	1.1	<0.001*
Random blood glucose (mg/dl)	133.1	40	96.9	24.8	0.002*
Hemoglobin (grams/deciliter)	11.15	1.02	11.12	1.67	0.954
White blood cells (*)	8.18	3.43	11.86	3.63	0.006*
Lymphocytes (%)	29.7%	12.2%	19.3%	8.1%	0.013*
Platelets (*)	220.6	97.5	230.1	92.3	0.78
			n(%)	n(%)	p-value**
Gravida	Nulli/Primi		6(46.2)	0(0.0)	-
	Multi		7(53.8)	21(100.0)	
Parity	Nulli/Primi		13(100)	8(38.1)	-
	Multi		0(0.0)	13(61.9)	
History of abortion	Yes		2(15.4)	5(23.8)	0.555
	No		11(84.6)	16(76.2)	
Blood groups & rh	A-		1(7.7)	2(9.5)	0.975
	A+		1(7.7)	3(14.3)	
	AB+		2(15.4)	3(14.3)	
	B+		4(30.8)	5(23.8)	
	O+		5(38.5)	8(38.1)	
Severity of corona	Mild		8(61.5)	13(61.9)	0.983
	Moderate/severe		5(38.5)	8(38.1)	
Mode of delievery***	vaginal		5(38.5)	1(4.7)	0.012
	Cesearean		8(61.5)	20(95.3)	
Admission to the critical care unit	Yes		0(0.0)	2(9.5)	-
	No		13(100)	19(90.5)	

SD - Standard deviation;

* Student's T-test;

** Pearson's chi-square test;

*** Significant at 0.05

In most of the study groups, 28 (82.4%) have operated on a cesarean section, the highest indication was histories of the previous scar representing half of the cases, followed by breach presentation (14.3%), elective cessearean section (10.7%), fetal distress (7.1%), cephalopelvic disproportion (7.1%), cord prolapse (7.1%), and the lowest was placenta accrete(3.6%) in just one woman. Regarding the peripartum period's parameters, there were no significant differences or associations between the placental histopathological findings groups. On the contrary, we can find that; more than one third of newborns with placental findings were getting delivered normally compared to about 5% of the other group (P=0.012). As shown in chart 2.

**Chart 2:** Comparison of fetal parameters and outcomes according to the presence of placental pathological findings

Parameters		Placental pathological findings				p-value a
		Yes (n=13)		No (n=21)		
		Mean	SD	Mean	SD	
Birth weight (Kg)		2.86	0.55	2.94	0.41	0.667
		n(%)		n(%)		p-value^b^
Apgar score (1^st^ min.)	<7	3(23.1)		8(38.1)		0.363
	7+	10(76.9)		13(61.9)		
Apgar score (5^th^ min)	<7	1(7.7)		2(9.5)		0.855
	7+	12(92.3)		19(90.5)		
Mode of delivery	Normal vaginal	5(38.5)		1(4.8)		0.012*
	Cesarean section	8(61.5)		20(95.2)		
Preterm	Yes	1(7.7)		8(38.1)		0.051
	No	12(92.3)		13(61.9)		
Stillbirth	Yes	1^c^(7.7)		2^d^(9.5)		0.855
	No	12(92.3)		19(90.5)		
Neonatal admission	Yes	3(23.1)		3(14.3)		0.513
	No	10(76.9)		18(85.7)		
Admission to the neonatal care unit	Yes	0(0)		3(14.3)		-
	No	13(100)		18(85.7)		

SD - Standard deviation;

a Student's T-test;

b Pearson's chi-square test;

* Significant at 0.05;

c this case presented at 36 weeks as severe oligohydramnios; Amniotic fluid index = 1cm, with a history of covid infection at 29 weeks;

d one of the cases had a previous scar, and her uterus ruptured during labor; the other case gave a history of midwife interference and had obstructed labor

The binary regression analysis revealed that; the period between the diagnosis of COVID-19 and the delivery significantly increased the odds of the presence of pathological findings by 2.75 (95% Confidence interval: 1.19 – 6.36) times for each week the patients getting infected earlier (Chart 3).

**Chart 3:** Logistic regression model for the predictive factors for placental pathological findings

Parameters	Correlation coefficient	p-value	Odds ratio	95% Confidence interval for odds ratio	
				Lower	Upper
COVID-19 Diagnosis to delivery (Weeks)	1.01	0.018*	2.75	1.19	6.36
White blood cells *10^3^	-0.04	0.884	0.96	0.54	1.70
Constant	-3.74	0.318	0.02		

* Significant at 0.05

## Discussion

Although there are no pathognomonic placental pathological features of COVID-19 infection, most of the researchers agreed that maternal vascular malperfusion, fetal vascular malperfusion, vasculitis and fibrinoid necrosis were the most common findings.^(1,8,11,15)^ In this study, only 13(38.2%) cases showed histopathological findings; including patients with fetal vascular malperfusion (76.9%) characterized by the abnormal variable size of fetal chorionic villi, 76.9% maternal vascular malperfusion (decidual arteriopathy, calcification and increase synciatail notes) which might be related to the hypercoagulable state caused by infection with COVID-19,^(16)^76.9% inflammatory pathology (lymphocytic villitis) findings that can be explained by the fact that COVID-19 is a viral infection and can induce inflammation, and villitis caused by the direct infection with some viruses, and only 53.8% thrombotic findings (fibrinoid necrosis of maternal arterioles) these may be attributed to the formation or deposition of thrombi in the placenta as a response to the virus.^(17)^

In similar studies concerning placental findings during COVID-19 infection Shanes et al.,^(11)^80% of COVID-19 cases had maternal vascular malperfusion implicating abnormalities in oxygen supply within the intervillous space leading to undesirable perinatal outcome, Singh et al.,^(18)^revealed enhanced syncytial knotting, higher microcalcifications, villous agglutination, and smaller fibrotic villi.

Regarding the indications of cs the highest indication was histories of the previous scar, and the lowest was placenta accreta in just one woman who was recently diagnosed with COVID-19 proved by PCR, we did not find COVID-19 spescific histopathological findings, instead we found chorionic villi adherent to the myometerum which is charactrisitic to placenta accrete this findings can be attributed to the fact that the infection was in late third trimester and near time of delivery.^(19,20)^

Regarding fetal distress as an indication of ceserean section, it was ranging from late decelartion and persistant bradycardia Many other researcher found that Higher incidence of abnormal CTG that mandate caesarean section in COVID-19 positive patients compared to control group.^(21,22)^

Possible determinants of COVID-19 related placental findings included lower white blood cell count in parallel with previous research findings reflecting that moderately symptomatic COVID-19 infections tend to cause lower white blood cell count,^(23)^higher random blood glucose levels resembling what has been found in other studies where this finding was likely to be associated with severe COVID-19 disease.^(24,25)^ The more severe the illness, the more likely it is to cause these laboratory abnormalities with associated histopathological findings in one or another body tissue like the placenta in this study's setting.

The other finding was the association between the pathological findings and lymphocytosis, but that comes in discrepancy with other research works where severe COVID-19 was associated with lymphopenia;^(26,27)^ this could be due to differences in the study sample properties relating to their immune response and health condition. None of the fetal parameters were found to be associated with histopathological placental findings.

Statistically, a significant association was found between the occurrence of placental findings and timing of COVID-19 infection during pregnancy and the duration of delivery from infection diagnosis; the earlier the infection and the longer the duration till delivery, the higher the likelihood of pathological findings. Even after regression analysis, the time duration between COVID-19 diagnosis and delivery was a potential determinant of histopathological findings in the placenta. There has been little or no research regarding this matter in previous literature owing to the recent history of this pandemic. These findings could be attributable to the long time needed for histopathological findings to develop following the infection.

The limited base of literature concerning this topic, fewer research resources for comparison and reasoning, and the relatively small sample size could be the study's limitations.also, not having a comparison group, of COVID-19 negative cases and also no cases during first trimester, In addition to not having viral molecular investigation in placental samples.

## Conclusion

A potential predictor for the occurrence of placental morphological abnormal findings following Covid-19 infection is the longer duration between the diagnosis of the infection and childbirth. Therefore, Covid 19 infection during late second and early third trimester of pregnancy rather than infection near term are more likely to inflect an abnormal change in the placental tissue. More research regarding this area is necessary, with larger sample size and a comparison group of COVID-19 negative cases.
